# Electrophoretically Snagging Viral Genomes in Wormlike Micelle Networks Using Peptide Nucleic Acid Amphiphiles and dsDNA Oligomers

**DOI:** 10.1021/acs.biomac.4c00332

**Published:** 2024-07-17

**Authors:** Kimberly Hui, Lingxiao Yan, James W. Schneider

**Affiliations:** Department of Chemical Engineering, Carnegie Mellon University, Pittsburgh, Pennsylvania 15213, United States

## Abstract

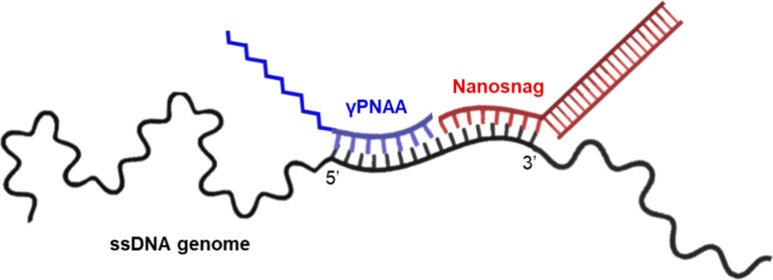

We demonstrate that the attachment of 30–170 bp dsDNA oligomers to ssDNA viral genomes gives a significant additional mobility shift in micelle-tagging electrophoresis (MTE). In MTE, a modified peptide nucleic acid amphiphile is attached to the viral genome to bind drag-inducing micelles present in capillary electrophoresis running buffers. Further attachment of 30–170 bp dsDNA oligomers drastically shifts the mobility of the 5.1 kB ssDNA genome of mouse minute virus (MMV), providing a new mechanism to improve resolution in CE-based analysis of kilobase nucleic acids. A model based on biased-reptation electrophoresis, end-labeled free-solution electrophoresis, and Ferguson gel-filtration theory is presented to describe the observed mobility shifts.

## Introduction

Historically, much of electrophoretic DNA analysis has been motivated by the need to obtain DNA sequence information by the length-based separation of restriction digests,^1^ short-tandem-repeat alleles,^2^ and DNA fragments generated by Sanger sequencing,^3^ among others. Recent advances in the large-scale production of biologics as well as viral and mRNA vaccines have provided new challenges for nucleic acid analysis, including the detection of trace microbial and biological contaminants in bioprocesses^4^ along with accurate titer of functional genomes in viral vaccines^5^ and of mRNA in lipid nanoparticles.^6^ Here, the nucleic acids of interest are, in most cases, partially folded, single-stranded (ss) DNA or RNA between 800 and 5000 nt in length. Each of these applications also requires discrimination of a target nucleic acid from other nucleic acids, including host-cell DNA and RNA, degraded viral or mRNA payloads, or distinct mRNA payloads in multivalent mRNA lipid nanoparticle formulations. Gel electrophoresis methods are capable of kilobase DNA or RNA quantitation,^7^ but typically have either poor resolution or a long runtime, depending on the platform used.

An alternative to gel electrophoresis is end-labeled free-solution electrophoresis (ELFSE), where nucleic acids have a negligibly charged protein, peptide, or polymer drag-tag chemically grafted to nucleic acids to be separated.^8^ This approach can provide high-resolution separations with fast runtimes, but due to constraints on the size and polydispersity of the drag-tag, is generally limited to separation of fragments less than 300 nt in length.^9^ We recently demonstrated that use of noncovalently attached nonionic micelles can extend the useful range of ELFSE to fragments above 500 nt.^10^ This “micelle-tagging electrophoresis (MTE)” method relies on the statistical fluctuation of micelle size to confer a uniform drag upon all fragments as required for high-resolution separations.^11,12^ This was accomplished by covalently grafting 18-carbon *n*-alkanes to PCR primers prior to their enzymatic extension to provide a binding site for micelles. We have also attached alkanes to DNA oligonucleotides by probe hybridization, giving a rapid separation of PCR products and rapid quantitation of several miRNAs in a closely related let-7 panel.^13,14^ In these applications, it was essential to use probes made of modified peptide nucleic acids (PNA)^15^ to give tight binding to targets and enable attachment of other oligonucleotides, when necessary. Much of the success of the MTE method relies on proper choice of micellar running buffer. In general, larger micelles provide a higher drag and are best suited to analysis of longer fragments. We have observed good results using various mixtures of C_*i*_E_*j*_-type nonionic surfactants, at concentrations of 1–3 wt % and viscosity of 1–2 cP. The low viscosity of separation buffers used helps circumvent difficulties encountered filling capillaries with more viscous gels, allowing for higher repeatability, efficiency, and convenience.

Here we show that MTE can be used to identify and quantitate mouse minute virus (MMV), a potential viral contaminant of interest to the bioprocessing community,^4^ by directly tagging the 5149-base ssDNA genome of MMV.^16^ Since the target molecule is longer than those previously used, we reformulated the C_*i*_E_*j*_ running buffer for better performance in this application. We also used a γ-modified version of PNA (γ^MP^PNA) that offers much higher melting transition temperatures (*T*_m_) than its unmodified PNA counterpart.^17,18^ γ^MP^PNA have a three-monomer ethylene oxide group attached to the γ carbon of the peptide backbone to confer this greater binding stability along with improved water solubility. Finally, we observed a dramatic sharpening of the product peak, with attendant decreased mobility, when further attaching short dsDNA segments in the vicinity of the γ^MP^PNA amphiphile. Attachment of this “nanosnag” induces an additional mobility shift that is dependent on the length of the nanosnag, suggesting a critical nanosnag length that is required to realize the full mobility shift. We propose a model for the migration of ssDNA tagged by γPNAA and nanosnags in this system, where migration is governed by independent free-volume filtration, ELFSE, and biased-reptation mechanisms. These results suggest that γPNAAs and nanosnags can be used to improve the resolving power of MTE for separation of long ssDNA and ssRNA, especially when samples are closely related in length.

## Materials and Methods

All reagents obtained from Sigma-Aldrich unless otherwise mentioned.

### γPNAA Synthesis, Purification, and Characterization

The γPNAA probe was synthesized manually using standard solid phase peptide synthesis protocols on a 10 μmol scale. Mini-PEG γPNA monomers were sourced from PNA Innovations (Woburn MA). The C-terminus was anchored to Fmoc Rink Amide MBHA resin (100 mg, 10% loading) and each monomer was coupled sequentially from the C-terminus to the N-terminus. During each coupling reaction, the Fmoc protection group was first removed from the terminal amine with 20% piperidine in *N*-methyl-2-pyrrolidone (NMP) (2 × 7 min). The next monomer (25 μmol) was mixed with 1-[bis(dimethylamino)methylene]-1*H*-1,2,3-triazolo[4,5-*b*]pyridinium 3-oxide hexafluorophosphate (HATU) (25 μmol) and *N,N*-diisopropylethylamine (DIEA) (10 μmol) for 2 min in 900 μL *N*-methylpyrrolidone (NMP) to activate the carboxylic acid before adding to the resin. Each coupling was stirred for 1 h at room temperature. The N-terminus was linked to a 5(6)-carboxyfluorescein dye conjugated to a lysine linker (AnaSpec), a three unit mini-PEG spacer (AnaSpec), and octadecanoic acid. Purification was done via HPLC with a symmetry C18 4.6 mm × 150 mm column (waters). The mobile phases were water (0.1% trifluoroacetic acid, TFA) and acetonitrile (0.1% TFA). Elution was performed with a 40 min linear gradient from 5 to 85% acetonitrile (0.1% TFA) at 1 mL/min. The masses of the products were verified on a PerSeptive Voyager STR MALDI-TOF mass spectrometer using α-cyano-4-hydroxycinnamic acid as the matrix. Following purification and characterization, the γPNAAs were dried by lyophilization and resuspended in water at a concentration of 20 μM.

### Viral Genome Extraction

Mouse minute virus (MMV) was purchased from ATCC (VR-1346). The nucleic acids from 200 μL of MMV stock was isolated using PureLink Viral RNA/DNA Mini Kit (Invitrogen), per the manufacturer’s protocol. This procedure is an enzyme-based extraction that uses Proteinase K to release nucleic acids from the viral particle. The genome was eluted from the column with 10 μL of DNase/RNase-free water (ThermoFisher). The nucleic acid yield from each extraction was determined by using a NanoDrop 2000c Spectrophotometer to be 150 ng/μL, or approximately 90 nM.

### Probe Hybridization and CE Detection

The extracted MMV genome was diluted to 1 nM in 10 v/v % formamide in DNase/RNase-free water. For analysis in MTE, 1 μL of a 20 μM stock solution of γPNAA probe was added to 15 μL of the diluted MMV sample. Two complementary DNA oligomers (Integrated DNA Technologies, Table 1) were annealed at 95 °C for 5 min and slow cooled to room temperature over 3 h to generate dsDNA nanosnags. For samples with two probes, 1 μL of a 20 μM DNA nanosnag solution was also added to the mixture. Probes were added at 1000-fold excess to ensure complete hybridization. All samples were heated to 60 °C for 5 min and then allowed to cool at room temperature for 5 min before MTE.

**Table 1:** γPNAA and Nanosnag Binding Sequences

probes	sequence (5′–3′)	length (binding portion)	binding site (bases)	predicted *T*_m_ (°C)
γPNAA	CAAAGAGCACGACGA	15	2484–2498	98
nanosnag	GGCCTATGATCAATACATCAAATCTGGAAA(PEG)_6_A_170_	30	2499–2528	59
poly-T complement	T_170_	0		65

Capillary electrophoresis (CE) experiments were performed on a P/ACE MDQ Plus (SciEx) equipped with laser-induced fluorescence (LIF) detection. LIF detection was performed at 488/520 nm excitation/emission. The capillary was a 50 μm ID fused-silica capillary (Polymicro Technologies) with length of 20 cm to the detector and 30 cm total length. Capillaries were prepared by flushing at 33 °C for 10 min with 10 v/v % PoP-6 polymer in 1× TBE at 20 psi to suppress electroosmotic flow. A running buffer of C_16_E_6_/C_12_E_5_/C_10_E_5_ (12:8:1 molar ratio) was prepared by first heating C_16_E_6_ to 50 °C to melt it, then adding an appropriate amount to 1× tris-borate-EDTA (TBE) at 40 °C, followed by shaking for 45 min at 40 °C and then for 45 min at 37 °C. C_12_E_5_ and C_10_E_5_ were then added to the buffer in appropriate amounts and the resulting suspension shaken for 1 h at room temperature. Before use in CE, buffers were centrifuged at 4000 rpm for 5 min to remove bubbles and capillaries were rinsed with the C_16_E_6_/C_12_E_5_/C_10_E_5_ (12:8:1 molar ratio) micelle buffer for 10 min at 20 psi. All electrophoresis buffers were prepared in 1× TBE buffer (pH 8.0). Samples were electrokinetically injected into the capillary with an applied voltage of 4 kV for 15 s. Electrophoretic separation was done in reverse polarity (injection at cathode, detection at anode) with an applied voltage of 20 kV at 30 °C. Data collection was performed using 32 Karat software.

### Capillary Viscometry

Viscosities of C_16_E_6_/C_12_E_5_/C_10_E_5_ (12:8:1 molar ratio) micelle buffer solutions ranging from 0.05 to 5.83 wt % were measured by capillary viscometry on a P/ACE MDQ Plus (SciEx) equipped with LIF detection. LIF detection was performed at 488/520 nm excitation/emission. The capillary was a 50 μm ID fused-silica capillary (Polymicro Technologies) with a length of 20 cm to the detector and 30 cm total length. The capillaries were filled with micelle buffer by rinsing for 10 min at 20 psi. A small plug of a 500 nM 5(6)-carboxyfluorescein in water was hydrodynamically injected into the capillary at 0.5 psi for 10 s. A pressure of 2 psi was used to hydrodynamically push the plug past the detector. The viscosity was determined using the elution time of fluorescein and assuming Hagen–Poiseuille plug flow.

## Results and Discussion

### PNAA Probe Design

**Figure 1:**
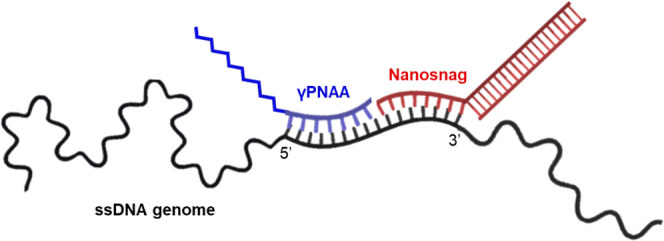
Schematic of the probes binding site on the MMV genome. The MMV genome is ssDNA and 5153 bases in length. γPNAA has a 15 base binding site. γPNA (not shown) has the same binding site and components as γPNAA, but without the 18-carbon alkane. The nanosnag has a 30-base binding site, binds adjacently to the 3′ end of γPNAA, and has a 30–170 bp dsDNA region. Because the nanosnag is not made of γPNA, it will not bind without assistance from coaxial stacking with the vicinal γPNAA.

We use two sequence-specific nucleic acid probes to tag the 5.1 kb ssDNA MMV genome for separation in MTE. A combination of PrimerBLAST and BLAST tools was used to identify a unique 15-base target sequence with high binding affinity and selectivity.^19,20^ With the sequence determined from this search, we synthesized both an unalkylated, but fluorescently labeled γPNA and a C18-alkylated γPNAA, each linked to fluorescein and a 3 unit mini-PEG linker. The nanosnag has an overhanging 30 base binding sequence to bind the MMV genome near the PNA binding site as shown in Figure 1. The longer 30-base region is required since DNA has a lower binding affinity for the MMV genome compared to γPNA.^17,21^ The nanosnag is targeted to bind directly adjacent to the 3′ end of the γPNA probes to take advantage of additional binding stabilization due to coaxial stacking.^22,23^ The 170 bp dsDNA portion of the nanosnag is made of poly A/T base pairs to avoid undesired off-target binding between the two probes.

Binding site numbers refer to the region of the MMV genome as reported on GenBank (J02275). The poly-T complementary oligomer was hybridized to the poly-A portion of the Nanosnag to form a 170 bp dsDNA oligomer. DNA/DNA *T*_m_ calculated using nearest-neighbor thermodynamics at 500 nM strand concentration in 89 mM NaCl. PNA/DNA *T*_m_ calculated using Giesen et al.^21^ model for PNA/DNA duplex stability. γPNA/DNA *T*_m_ estimated with an addition of 2 °C per substitution as observed by Sahu et al.^18^

### Micelle Running Buffer

The micelle buffer was a ternary mixture of C_16_E_6_, C_12_E_5_, and C_10_E_5_ surfactants at a 12:8:1 molar ratio, which was formulated to generate long micelles. Increased molar ratios of C_16_E_6_ and C_12_E_5_ were used to elongate micelles, while C_10_E_5_ served as an “end-capper” to help solubilize and control the micelle length. Micelles made from similar suspensions have been reported to range from 1800 to 3400 nm in length.^24,25^ As surfactant concentration increases, micelles will grow in size and in number. Above the overlap concentration (c*), the wormlike micelles no longer exist as individual hydrodynamically segregated micelles in solution but instead they overlap to form a network of interconnected pores.^26^c* was determined to be 0.47 wt % by measuring the viscosities of various dilutions of the micelle buffer and determining the intersection of two linear fits representing the high and low viscosity regimes from a log–log plot. Above c*, the pore size can be approximated by the hydrodynamic correlation length ξ_H_, which is related to the collective diffusion coefficient of a micelle network. We estimate the pore size of our system is approximately 15 nm at 0.97 wt % surfactant using ξ_H_ and a correction factor for C_*i*_E_*j*_ surfactants reported by Kato et al.^27^

### Micelle-Tagging Electrophoresis

**Figure 2:**
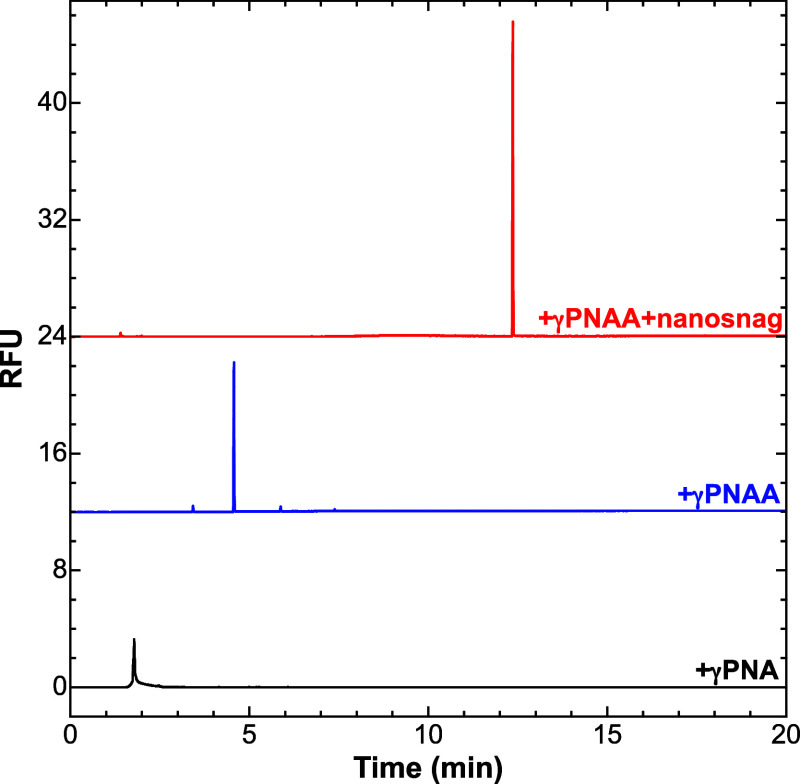
Electropherograms showing the MMV genome bound to γPNA, γPNAA, and both γPNAA and a 170 bp nanosnag. MTE separation was done in 0.97 wt % surfactant buffer at 30 °C. CE Conditions: applied voltage 20 kV, capillary length 30 cm, length to detector 20 cm.

Each of the probes (γPNA, γPNAA, and γPNAA + nanosnag) was hybridized to the ssDNA genome and separated in MTE, using a 0.97 wt % micelle buffer. Electropherograms showing the effects each of the probes have on the elution behavior are presented in Figure 2. The first trace shows the ssDNA genome bound to γPNA, where the probe acts only as a fluorophore for detection and the bound genome quickly elutes in 1.7 min. We refer to the ssDNA genome bound to γPNA as naked ssDNA since γPNA binding is not expected to affect the electrophoretic mobility of the MMV genome. However, binding the end-alkylated γPNAA probe to the genome not only shifts the elution to 4.8 min, but also results in a marked increase in LIF signal due to the attachment of the alkane. Upon binding of the nanosnag, the complex undergoes another mobility shift, now eluting at 11.2 min with a further jump in LIF signal intensity.

The increase in signal intensity associated with probe binding in MTE as an example of sample stacking.^28^ Sample stacking is an online concentration strategy where large amounts of dilute samples are injected and concentrated into a short zone, thus increasing the concentration and signal strength. Stacking occurs when a sample migrates from a region of high velocity into a region of slow velocity, such that the concentration must increase due to sample accumulation at that boundary. Mass conservation dictates that the degree of concentration and subsequent signal enhancement is proportional to the ratio of the initial velocity and the final velocity, in theory.^28^

We define the stacking efficiency (SE) as the ratio of the peak width (at half-maximum) in the absence of surfactant and the peak width in the presence of surfactant. In the case of ssDNA + γPNAA, SE is 6× greater than that for naked ssDNA, while the velocity decrease is about 5×, based on the electrophoretic mobility of each in the absence of surfactant. Some further increase in SE could be traced to a suppression of axial diffusion by micelle binding, but this was not studied in detail.

**Figure 3:**
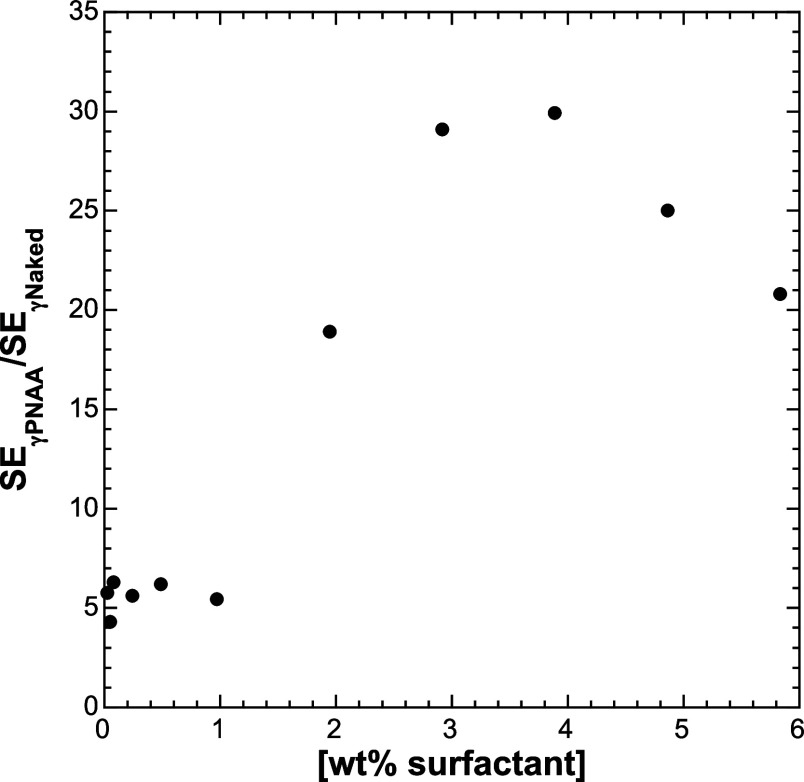
Ratio of SE_γPNAA_/SE_Naked_ as a function of surfactant wt %. Increase in ratio corresponds to the formation of an entangled micelle network.

Figure 3 shows the ratios of SE for naked ssDNA vs ssDNA + γPNAA across a range of surfactant concentrations from 0.05 to 5.8 wt %. There is a dramatic increase in the SE ratio above c*, suggesting that the presence of a weakly overlapping network of micelles is required for maximum stacking. The downturn in SE at the highest two concentrations is likely due to the high degree of entanglement and high viscosities of the buffers used, which leads to peak smearing and a shifted baseline following the peak from material potentially trapped in the micelle matrix.

### Electrophoretic Mobility Shifts

**Figure 4:**
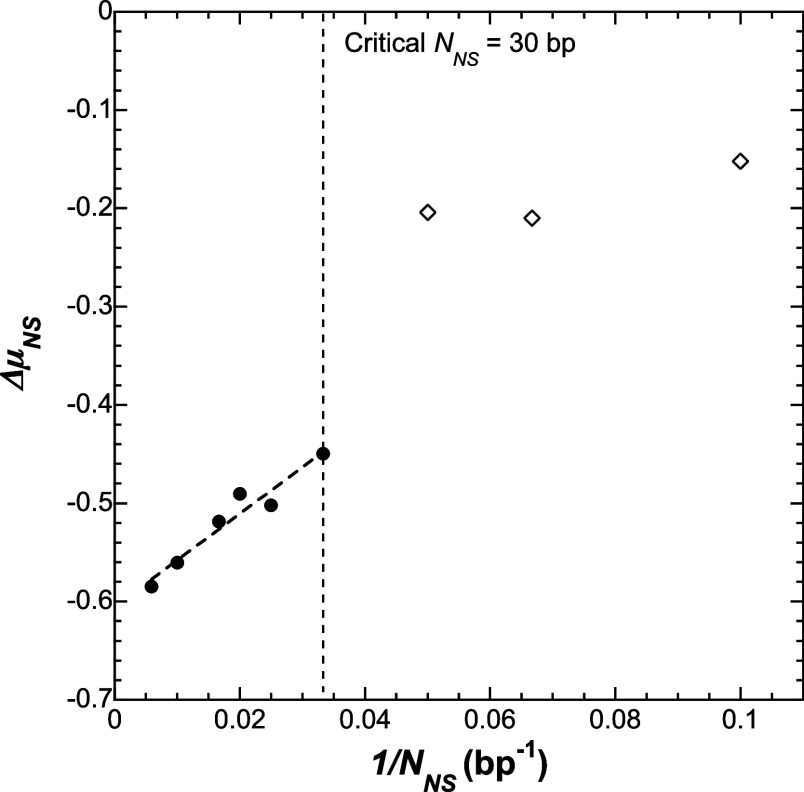
BRM plot of Δμ_NS_ vs 1/*N*_NS_. Above *N*_NS_ = 30 bp, there is a linear relationship which is indicative of a biased-reptation mechanism. Linear Fit: *y* = 4.7*x* – 0.6, *R*^2^ = 0.94.

The dsDNA nanosnag is short (170 bp) compared to the ssDNA MMV viral genome (5149 nt) but its attachment to the MMV genome has a substantial impact on the mobility of the MMV genome. In fact, even shorter nanosnags impart significant mobility shifts down to lengths of 30 bp as shown in Figure 4. Here we tested different lengths of dsDNA in nanosnags (*N*_NS_ = 10, 15, 20, 30, 40, 50, 60, 100, 170 bp) using a 0.97 wt % micelle buffer. The mobility remains relatively constant when short nanosnags are attached but undergoes a large mobility shift once *N*_NS_ reaches a critical length of about 30 bp. Above 30 bp, the mobility of the complex continues to decrease as LNS increases. In Figure 4 the mobility shift experienced when the γPNAA-tagged genome is further tagged with a nanosnag (Δμ_NS_) is plotted versus the reciprocal of the length of the nanosnag, demonstrating a linear dependence between the two as predicted by the biased-reptation model (BRM) for gel electrophoresis.^29^ However, in our case it is the length of the nanosnag that sets the agreement with the BRM. Additionally, there is only a small shift when using nanosnags less than 30 bp in length.

**Figure 5:**
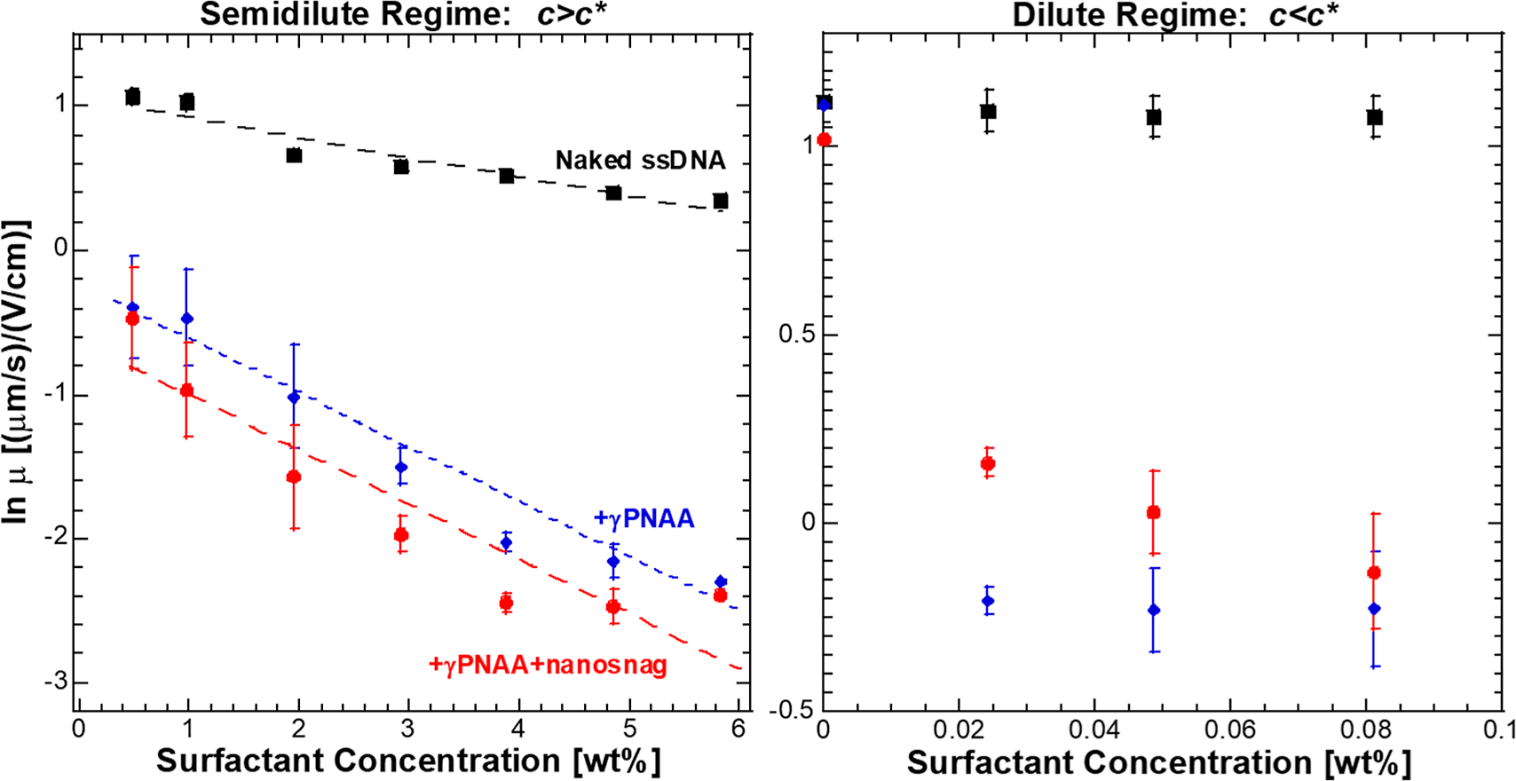
Ferguson plots of ln μ vs surfactant concentration [wt %] for the viral ssDNA genome bound to γPNA (black), to γPNAA (blue), and both γPNAA and a 170 bp nanosnag (red). The slope and intercept of each linear fit were used to determine *K*_R_ and μ_0_, respectively, with eq 6. Values are listed in Table 2. Error bars represent standard deviation (*n* = 3). CE conditions: applied voltage 20 kV, capillary length 30 cm, length to detector 20 cm.

To better determine the mechanisms of the mobility shifts, we measured the mobilities of γPNA-tagged, γPNAA-tagged, and (γPNAA + nanosnag)-tagged MMV genomes at increasing concentrations of surfactant as shown in Figure 5. In each case, the variously tagged MMV appear to fit the Ferguson gel-filtration model,^30^ with
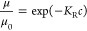
1where *K*_R_ is a retardation coefficient that is roughly proportional to the radius of gyration of the electrophoresing polyampholyte, *c* is the total surfactant concentration, and μ_0_ is the free-solution mobility of the MMV genome; that is, in the absence of surfactant. Each of the three complexes appears to have a different μ_0_, which is not expected since the attachment of these small peptides and/or oligomers should have a negligible effect in the ratio of charge to friction for the MMV genome in the absence of surfactant. These differences in apparent μ_0_ have to do instead with the micelle-tagging and biased reptation of the attached nanosnag.

To account for the strong dependence of tagged genome mobility on the nanosnag length, we have developed a model to predict it using BRM theory. Following the approach of Semenov et al.,^29,31^ we first balance the drag force of the micelle- and nanosnag-tagged MMV ssDNA genome with the electric force driving its motion
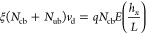
2where ξ is the friction coefficient for a blob of the DNA fragment, *N*_cb_ is the number of charged blobs in the complex, *N*_ub_ is the number of uncharged blobs in the complex (here, micelles are represented by a hydrodynamically equivalent polymer), *v*_d_ is the velocity of the nanosnag through its confining pore, *q* is the charge per blob, *E* is the electric field in the *x*-direction (axial direction, from anode to cathode), *h*_*x*_ is the end-to-end distance of the nanosnag projected in the *x* direction, and *L* is the contour length of the nanosnag. We introduce a parameter α, which is the drag of the attached micelle expressed as the number of charged blobs that would have the same drag as a theoretical uncharged blob that stands in for a micelle. Solving for *v*_d_, we have
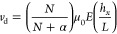
3We note that the free solution mobility of unmodified DNA, μ_0_ = *q*/ξ. In the above analysis we have neglected the charge contribution of the short dsDNA nanosnag, which is much shorter than the MMV genome. The velocity in the axial direction *ẋ* is obtained by resolving *v*_d_ in the axial direction
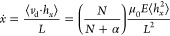
4

Assuming Gaussian statistics for the nanosnag motion, the mean-square end-to-end distance of the nanosnag, projected in the *x* direction is given by (1/3)*Ll*_k,NS_, and recognizing that *ẋ*/*E* = μ, we have
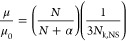
5

Finally, we account for the Ferguson gel-filtration of the MMV genome by multiplying the right-hand side of eq 5 by exp (*−K*_R_*c), which is equal to the fraction of free volume available to electrophoresing DNA of a given size. Equation 6 is the resulting mobility equation for the micelle- and nanosnag-tagged genome mobility, represented in logarithmic form
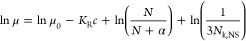
6which provides a convenient way to plot mobility data and assess the impact of each process. To obtain parameters, we fit the experimental mobility data for each of the probes to eq 6. This is shown in Figure 5, with each data set appearing to be linear. The parameters determined from the fit are listed in Table 2. The contour length of the critical nanosnag (*N*_NS_ = 30 bp) is approximately 10 nm, which is near the pore size in the micelle matrix (about 15 nm). This implies that when the nanosnag reaches that critical length, it becomes trapped in a series of pores and must reptate out of its confining pores one by one. The nanosnag therefore compels the entire MMV genome to follow the pore-to-pore transit of the attached nanosnag.

**Table 2:** Free-Solution Mobilities, Friction Coefficients, and Micelle Sizes from Fit of Figure 5 to eq 6 (Length of Nanosnag, *L*_NS_ = 170 bp)^a^

probes	apparent μ_0_ [(μm/s)/(V/cm)]	*K*_R_	α	*R*_g_ (nm)
naked ssDNA	3.09	0.14		
+γPNAA	0.78	0.38	15300	40
+γPNAA +nanosnag	0.54	0.38	11500	30

a *R*_g_ was estimated using the Kratky–Porod model of eq 7.

The free solution mobility (μ_0_, that observed in the absence of a separating matrix) is easily observed in the limit of *c* → 0. While we expect all three cases (naked, +γPNAA, and +γPNAA +nanosnag) to have the same value as *c* → 0, there were not enough data collected below 1% surfactant to give a confident extrapolation in that limit for the +γPNAA, and +γPNAA +nanosnag cases. Still, a zoom-in on the dilute concentration data in Figure 5 show the curves bending to the expected limit for the (+γPNAA +nanosnag) case. This shape reflects a weaker partitioning to the micelle phase in the dilute regime, with attendant loss of micelle-induced drag. We note that the (+γPNAA +nanosnag) case appears to deviate from linearity at higher concentrations, which may be due to steric interference of the vicinal nanosnag with the *n*-alkane group on the PNAA to weaken the partitioning. The value extrapolated from the naked ssDNA (3.09 (μm/s)/(V/cm)) compares well to values in literature, which have been reported to be in the range of 3.0–3.4 (μm/s)/(V/cm) for ssDNA.^32,33^

When the γPNAA probe is attached, the mobility extrapolated to zero surfactant concentration (μ_MTE_) represents the hypothetical free-solution mobility of the ssDNA genome with a micelle drag tag attached. The approximate size and friction of the micelles attached to the ssDNA thus can be deduced from μ_MTE_ and eq 6. This analysis yielded α = 15,300, which is higher than previously reported values for C_*i*_E_*j*_ surfactant micelles.^10^ The equivalent radius of gyration (*R*_g_) for the attached surfactant material can be estimated assuming chain statistics for a hypothetical ssDNA segment of length α, which has the same drag as the attached material. Following Desruisseaux et al.,^34^ we have
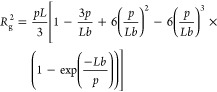
7where *p* is the persistence length, *b* is the monomer length, and *L* is the number of bases. Using *p* ≈ 0.75 nm and *b* ≈ 0.43 nm for ssDNA, eq 7 gives *R*_g_ = 40 nm for the attached micelle drag-tag in the case of +γPNAA and *R*_g_ = 30 nm for the +γPNAA +nanosnag case. The lower value may be due to interference of the nanosnag with micelle attachment to the nearby *n*-alkyl group of the γPNAA. We note that use of eq 6 required an estimation of the number of Kuhn lengths in the nanosnag; we used *l*_k_ = 100 nm for the dsDNA nanosnag to yield *N*_k,NS_ = 1.7.

The fitted retardation coefficient (*K*_R_) is about 2.7× higher for both the (+γPNAA +nanosnag) cases compared to the naked ssDNA. This is expected as *K*_R_ scales with *R*_g_^*2*^ so that the free volume decreases on attachment of a micelle, leading to a steeper slope. Interestingly, the further attachment of a nanosnag does not impact *K*_R_ since the slopes are nearly the same in the +γPNAA and (+γPNAA +nanosnag) cases. Attachment of the smaller nanosnag has no measurable impact on the gel-filtration properties of the micelle-tagged ssDNA.

We note that while the conformation of the short dsDNA nanosnag is likely not well approximated by Gaussian statistics, it is possible that the transit of the nanosnag through the network of pores allows it to sample many orientations during elution of the tagged genome, and this angle-averaging could give rise to a similar value for ⟨*h*_*x*_^2^⟩ in eq 4. Experiments with varying ssDNA lengths, possibly as digests of MMV, may be useful in better determining the role of the nanosnag in redirecting the electrophoresis of the attached genome. These experiments could also help determine if the BRM theory is the best approach for describing the mobility shifts observed in this work.

## Conclusions

We have shown that stiff dsDNA oligomers (nanosnags) can induce major shifts in electrophoretic mobility in wormlike micelle networks when used in conjunction with MTE. Electrophoretic migration is governed by three independent mechanisms–filtration through a surfactant micelle sieving matrix, tagging by a micelle drag tag, and snagging of short dsDNA nanosnags in a pore. The mobilities can be predicted using a modified Ferguson equation, with terms accounting for the micelle drag and nanosnag reptation. Elution time can be easily tuned by surfactant concentration and attachment of probes to suit the needs of a specific assay, such as for faster analysis times or higher resolution.

This effect can expand the lengths of ssDNA and ssRNA that MTE can separate, enabling high resolution separation of long kb-length samples that are closely related in length by attachment of a γPNAA or nanosnag. We envision many potential applications for this new separation technique, especially with the rise of nucleic acid–based therapeutics such as mRNA vaccines. This method is rapid, easily scalable, and requires little solvent and resources, making it a promising method for quality control in manufacturing.

## Supporting Information Available

Chemical structures, control experiments, and electropherograms used to generate Figures 3–5 are available as Supporting Information (PDF)
