# Supplementary material for: Electrophoretically Snagging Viral Genomes in Wormlike Micelle Networks Using Peptide Nucleic Acid Amphiphiles and dsDNA Oligomers
Source: Biomacromolecules. 2024 Jul 17;25(8):4891–7. doi: 10.1021/acs.biomac.4c00332 (PMC11322999; doi:10.1021/acs.biomac.4c00332)
Supplement: Supplementary file 1 — bm4c00332_si_001.pdf [file bm4c00332_si_001.pdf]

Supporting Information: Electrophoretically snagging viral genomes in wormlike micelle networks using peptide nucleic acid amphiphiles and dsDNA oligomers

Kimberly Hui, Lingxiao Yan, and James W. Schneider*

Department of Chemical Engineering, Carnegie Mellon University, Pittsburgh PA 15213 USA

*Email: schneider@cmu.edu

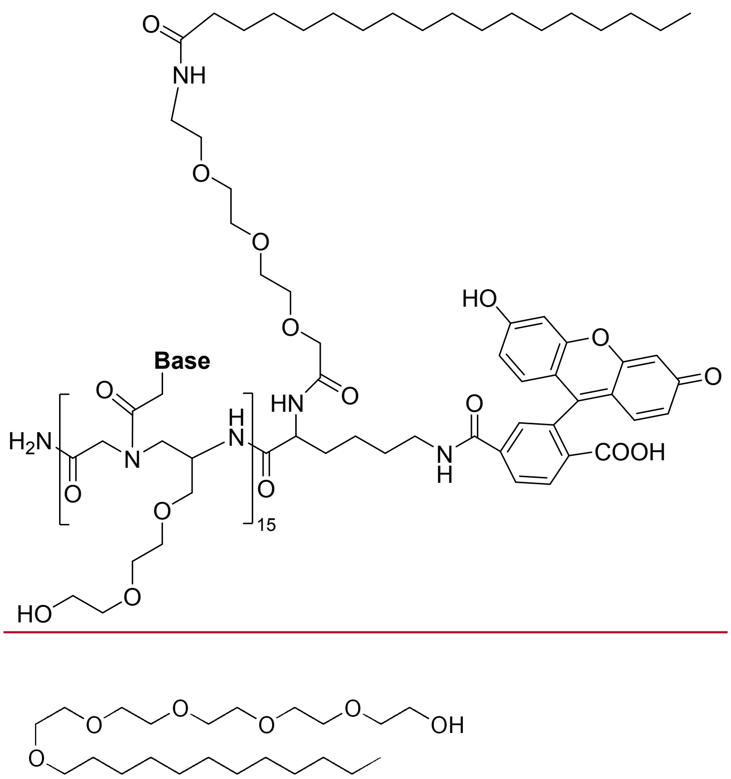

Figure S1. Chemical structure of γPNAA (3’→ 5’ sense from left to right). “Base” refers to nucleobase (adenine, thymine, guanine, cytosine) along with C12E5 (pentaethylene glycol monododecyl ether)

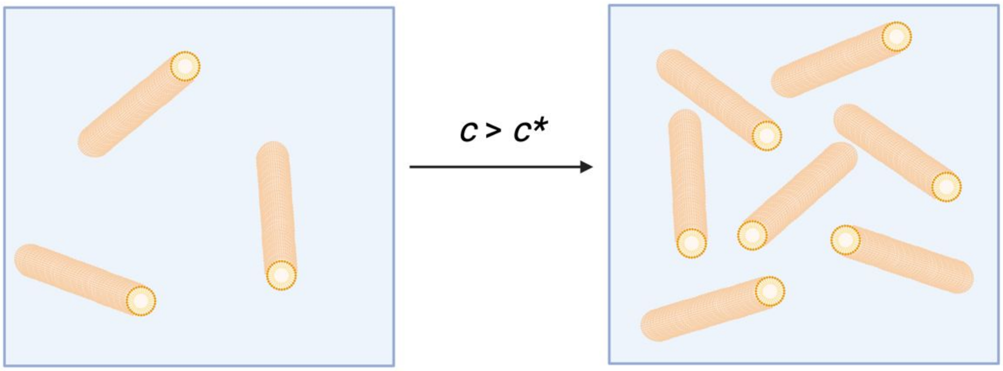

Figure S2. Depiction of micelle structure above and below the overlap concentration (c^*). Created with BioRender.com.

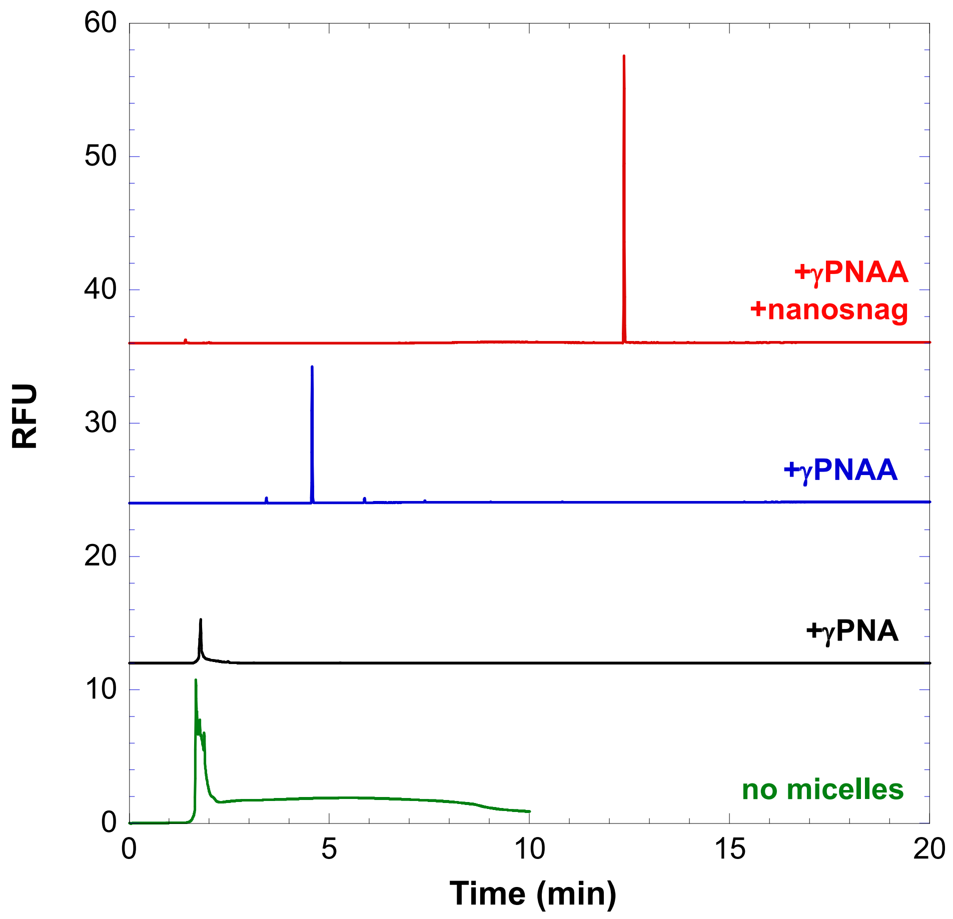

Figure S3. Electropherograms showing the MMV genome bound to γPNA, γPNAA, and both γPNAA and a 170 bp nanosnag. MTE separation was done in 0.97 wt% surfactant buffer at 30°C. CE Conditions: Applied voltage – 20 kV, Capillary length – 30cm, Length to detector – 20 cm. An electropherogram is also included for the γPNAA elution in 1x TBE (pH 8.0) buffer without micelles for comparison.

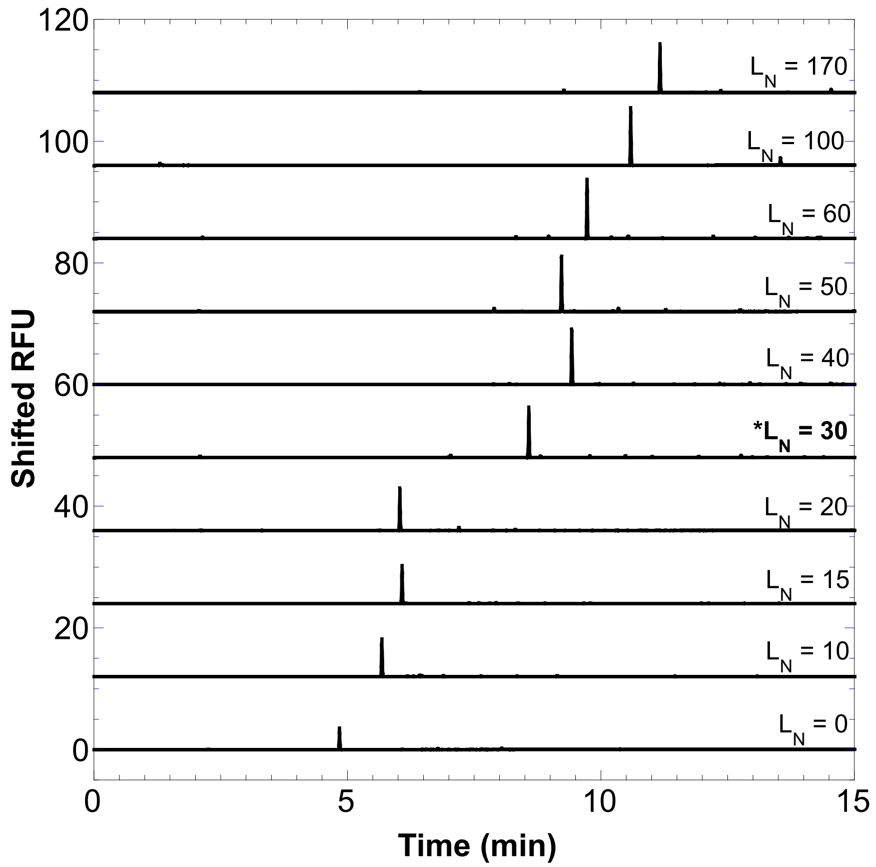

Figure S4. Electropherograms of MMV genome bound to γPNAA and nanosnags, ranging in length (L_N) from 10 to 100 bp, in 0.97 wt% surfactant buffer. The critical nanosnag length (*L_N) is 30 bp, which is the nanosnag length needed to generate a mobility shift. CE Conditions: Applied voltage – 20 kV, Capillary length – 30cm, Length to detector – 20 cm. Data arbitrarily shifted on the y-axis for comparison purposes.

Table S1. Compositions of the Buffers Used in Figures S3 and S4.

	**Buffer Composition (mM $C_{16}E_6$/mM $C_{12}E_5$/mM $C_{10}E_5$)**	***c/c****	***wt%***	**Relative Viscosity [η/η_o]**
i	0.3/0.2/0.025	0.05	0.052	1.0
ii	0.6/0.4/0.05	0.10	0.049	1.0
iii	1/0.67/0.083	0.17	0.08	1.0
iv	3/2/0.25	0.51	0.24	1.2
v	6/4/0.5	1.03	0.49	1.5
vi	12/8/1	2.05	0.97	3.2
vii	24/16/2	4.10	1.9	9.4
viii	36/24/3	6.15	2.9	19.9
ix	48/32/4	8.20	3.9	33.6
x	60/40/5	10.3	4.9	52.3
xi	72/48/6	12.3	5.8	75.6

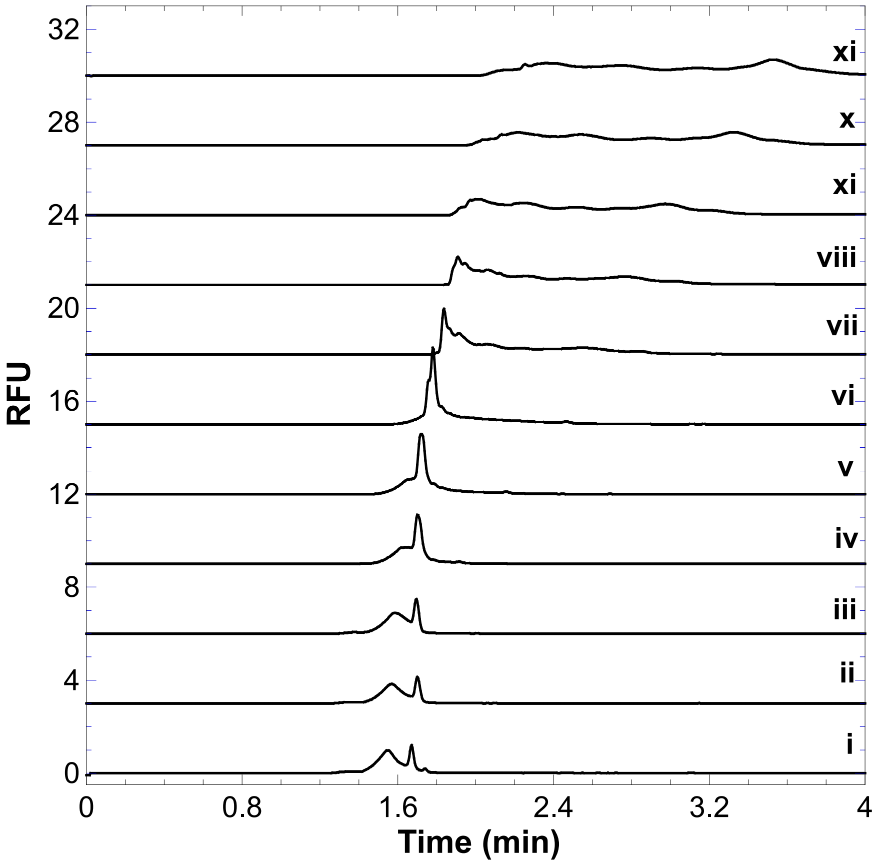

Figure S5. Electropherograms of MMV genome bound to unalkylated γPNA probe, at surfactant concentration from 0.05 to 5.8 wt% (Table S1). CE conditions: Applied voltage – 20 kV, Capillary length – 30cm, Length to detector – 20 cm.

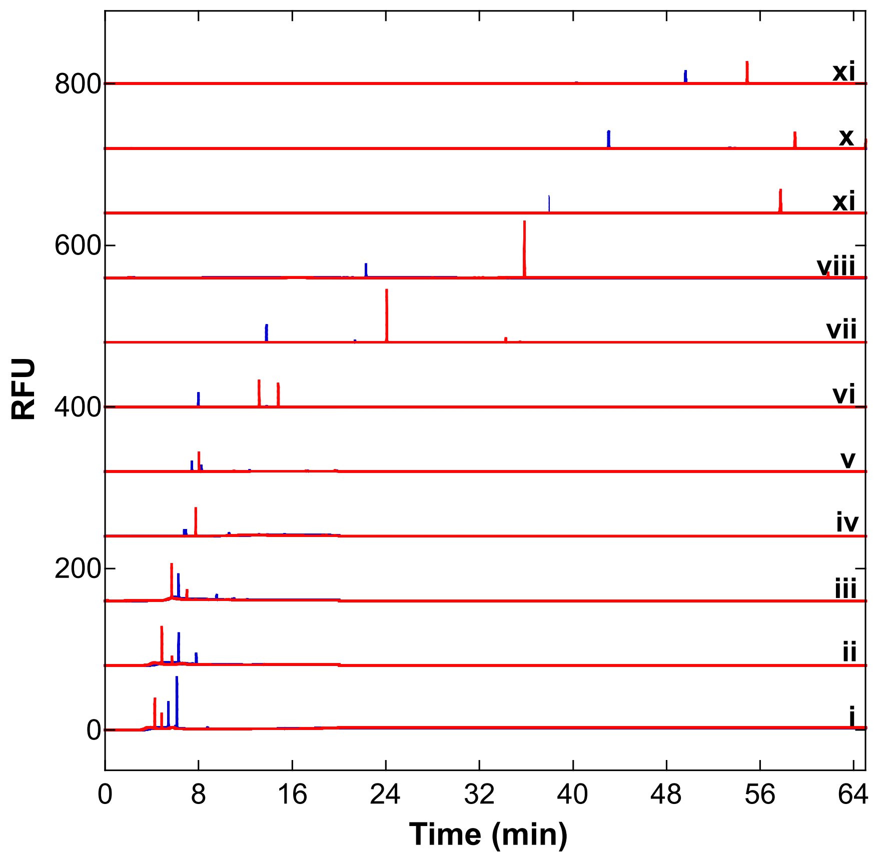

Figure S6. Electropherograms of MMV genome bound to γPNAA (blue) and both γPNAA and nanosnag (red), at surfactant concentrations ranging from 0.05 to 5.8 wt% (Table S1). CE conditions: Applied voltage – 20 kV, Capillary length – 30cm, Length to detector – 20 cm

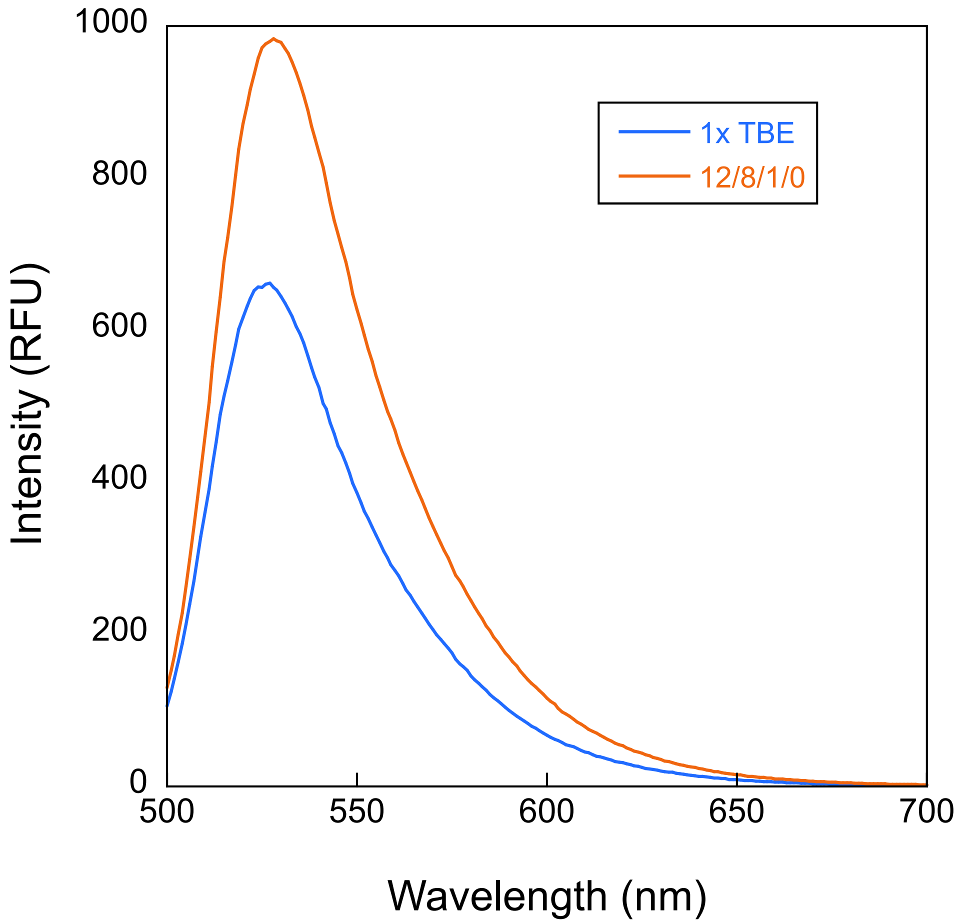

Figure S7. Fluorescence emission spectra for PNA amphiphile in 1x TBE and in the MTE running buffer (12 mM C16E6, 8 mM C12E5, 1 mM C10E5). Concentration – 1 µM; Excitation wavelength = 488 nm.
